# Complete Genome Sequence of the Novel Temperate *Clostridium difficile* Phage phiCDIF1296T

**DOI:** 10.1128/genomeA.00839-15

**Published:** 2015-08-20

**Authors:** Johannes Wittmann, Thomas Riedel, Boyke Bunk, Cathrin Spröer, Sabine Gronow, Jörg Overmann

**Affiliations:** a Leibniz Institute DSMZ-German Collection of Microorganisms and Cell Cultures, Braunschweig, Germany; b German Centre of Infection Research (DZIF), partner site Hannover-Braunschweig, Braunschweig, Germany

## Abstract

*Clostridium difficile* contains many integrated and extrachromosomal genetic elements. In this study, we determined, annotated, and analyzed the complete genome of the *C. difficile* bacteriophage phiCDIF1296T using single-molecule real-time sequencing technology. To our knowledge, this represents the largest genome (131 kb) of a temperate *C. difficile* phage recognized so far.

## GENOME ANNOUNCEMENT

In this study, we determined the complete genome sequence of the bacteriophage phiCDIF1296T *de novo* using single-molecule real-time (SMRT) sequencing technology. The extrachromosomal phage genome sequence was detected during a whole-genome sequencing approach with the *Clostridium difficile* strain DSM 1296^T^.

Complete genome sequencing was carried out using the PacBio *RSII* (Pacific Biosciences, Menlo Park, CA). Assembly was performed using the RS_HGAP_Assembly.3 protocol included in SMRT Portal version 2.2.0 and yielded the complete chromosome of phage phiCDIF1296T. This sequence was circularized and adjusted to the putative small subunit of the terminase gene as position 1 (CDIF1296T_phi001) of the phage chromosome. A final genome quality of QV60 was determined during resequencing using the RS_BridgeMapper.1 protocol in SMRT Portal. Automated genome annotation was performed using Prokka (1), followed by manual curation.

Phage phiCDIF1296T has a genome of 131,326 bp, with a coding percentage of 86.4% and a G+C content of 26.4%. To our knowledge, this represents the largest genome of a temperate *C. difficile* phage recognized so far (2). We predicted 181 unique coding sequences, of which 59 revealed a conserved domain at the amino acid level after BLASTp analysis. Fifty genes were assigned a predicted function. Analysis with tRNAscan-SE 1.21 (3) revealed one predicted tRNA for serine. The complete phage genome could be divided into functional clusters that encode DNA packaging, head and tail morphogenesis, host cell lysis, and replication. We identified genes for the large terminase subunit (CDIF1296T_phi002), a minor tail protein (CDIF1296T_phi024), two putative tail fibers (CDIF1296T_phi032/35), two tail components with a putative enzymatic activity (CDIF1296T_phi029/030), and a tail tape measure protein (CDIF1296T_phi025).

In addition, we detected genes encoding proteins that confirm the temperate character of phiCDIF1296T, including a recombinase (CDIF1296T_phi046), integrase (CDIF1296T_phi052), two putative transposases (CDIF1296T_phi054/148), repressors (CDIF1296T_phi048/051/154), antirepressors (CDIF1296T_phi022/129), and a large number of 12 putative transcriptional regulators (phiCDIF1296T_020/047/048/051/131/133/138/153/155/167/171/172).

The gene cluster for host cell lysis consisted of one gene for an *N*-acetylmuramoyl-l-alanine amidase (CDIF1296T_phi042), glucosaminidase (CDIF1296T_phi043) and two putative holin-like proteins (CDIF1296T_phi044/055) with one and two transmembrane domains (class II holin), respectively (4).

Genes for DNA replication encoded a putative Holliday junction resolvase (CDIF1296T_phi057), a DNA primase (CDIF1296T_phi071), a helicase (CDIF1296T_phi072), a nuclease (CDIF1296T_phi062), an Erf-like protein (CDIF1296T_phi074), a DNA ligase (CDIF1296T_phi115), and a DNA polymerase (CDIF1296T_phi084). Additionally, phage phiCDIF1296 harbored three genes coding for methylases that are probably involved in DNA modification (CDIF1296T_phi060/061/087). Furthermore, we identified genes that encode proteins for DNA metabolism, such as guanylate kinase (CDIF1296T_phi058) and dUTPase (CDIF1296T_phi056).

Finally, we determined several additional interesting genes, e.g., for a putative anaerobic ribonucleoside-triphosphate reductase that might play a role in nucleotide metabolism (CDIF1296T_phi113), a putative antirestriction protein that might protect the phage genome against the host restriction system (CDIF1296T_phi166), and a Doc-like protein that might act as a prophage maintenance system killer protein (CDIF1296T_phi145) (5).

### Nucleotide sequence accession numbers.

The complete genome is deposited at GenBank under the accession no. CP011970. The version described in this paper is version CP011970.1.
